# MMP9 but Not EGFR, MET, ERCC1, P16, and P-53 Is Associated with Response to Concomitant Radiotherapy, Cetuximab, and Weekly Cisplatin in Patients with Locally Advanced Head and Neck Cancer

**DOI:** 10.1155/2009/305908

**Published:** 2009-12-29

**Authors:** George Fountzilas, Anna Kalogera-Fountzila, Sophia Lambaki, Ralph M. Wirtz, Angelos Nikolaou, Georgia Karayannopoulou, Mattheos Bobos, Vassiliki Kotoula, Samuel Murray, Alexandros Lambropoulos, Gerasimos Aravantinos, Konstantinos Markou, Eleni Athanassiou, Despina Misailidou, Konstantine T. Kalogeras, Demosthenis Skarlos

**Affiliations:** ^1^Department of Medical Oncology, “Papageorgiou” Hospital, Aristotle University of Thessaloniki School of Medicine, Thessaloniki, Greece; ^2^Department of Radiology, “AHEPA” Hospital, Aristotle University of Thessaloniki School of Medicine, Thessaloniki, Greece; ^3^Siemens Healthcare Diagnostics, Cologne, Germany; ^4^Department of Otorhinolaryngology, “AHEPA” Hospital, Aristotle University of Thessaloniki School of Medicine, Thessaloniki, Greece; ^5^Department of Pathology, Aristotle University of Thessaloniki School of Medicine, Thessaloniki, Greece; ^6^Department of Molecular Biology and Genetics, “Metropolitan” Hospital, Piraeus, Greece; ^7^Laboratory of Molecular Biology, Department of Obstetrics and Gynecology, “Papageorgiou” Hospital, Aristotle University of Thessaloniki School of Medicine, Thessaloniki, Greece; ^8^Third Department of Medical Oncology, “Agii Anargiri” Cancer Hospital, Athens, Greece; ^9^Department of Radiation Oncology, “Agios Savvas” Cancer Hospital, Athens, Greece; ^10^Department of Radiation Oncology, “Papageorgiou” Hospital, Thessaloniki, Greece; ^11^Hellenic Cooperative Oncology Group (HeCOG), Data Office, Athens, Greece; ^12^Second Department of Medical Oncology, “Metropolitan” Hospital, Piraeus, Greece

## Abstract

Concomitant administration of radiotherapy with cisplatin or radiotherapy with cetuximab appear to be the treatment of choice for patients with locally advanced head and neck cancer. In the present retrospective analysis, we investigated the predictive role of several biomarkers in an unselected cohort of patients treated with concomitant radiotherapy, weekly cisplatin, and cetuximab (CCRT). We identified 37 patients treated with this approach, of which 13 (35%) achieved a complete response and 10 (27%) achieved a partial response. Severe side effects were mainly leucopenia, dysphagia, rash, and anemia. Tumor EGFR, MET, ERCC1, and p-53 protein and/or gene expression were not associated with treatment response. In contrast, high MMP9 mRNA expression was found to be significantly associated with objective response. In conclusion, CCRT is feasible and active. MMP9 was the only biomarker tested that appears to be of predictive value in cetuximab treated patients. However, this is a hypothesis generating study and the results should not be viewed as definitive evidence until they are validated in a larger cohort.

## 1. Introduction

Concomitant chemo-radiotherapy, mainly with cisplatin is the standard combined modality approach for the treatment of patients with locally advanced squamous cell carcinoma of the head and neck (SCCHN) region, because it prolongs survival and increases the chance of organ preservation compared to radiotherapy (RT) alone [1–3]. Several potential mechanisms, through which cisplatin acts as a radiosensitizer, have been reported reviewed in [4].

 Single-agent cisplatin (100 mg/m^2^) administered every 3 weeks concomitantly with RT is widely used since this high dose confers a systemic effect and at the same time acts as a radio-sensitizer [5]. However, the therapeutic benefit derived from the combined modality is counterbalanced in many cases by prohibitive toxicity, mainly neurotoxicity, ototoxicity, emesis, and stomatitis [6]. In order to reduce cisplatin-related toxicity, several investigators tested alternative schedules of cisplatin administration, such as daily or weekly infusions. The use of these different schedules is supported by in vitro data showing that low doses of cisplatin and RT, when combined, act synergistically in cell killing [3]. During the last few years, investigators within the Hellenic Cooperative Oncology Group (HeCOG) had adopted the weekly schedule of cisplatin concomitantly with RT for the treatment of patients with locally advanced SCCHN [7].

 It is well documented that epidermal growth factor receptor (EGFR) is overexpressed in 42% to 80% of SCCHN cases [8, 9]. EGFR plays a pivotal role in proliferation and survival of SCCHN cells and its overexpression is associated with advanced stages and poor outcome [10, 11]. In previous studies EGFR expression was proposed as an even stronger predictor of locoregional control than T stage [9]. For this reason EGFR appears to be an attractive target of anticancer drugs. Furthermore, EGFR is an important determinant of response to RT and confers protection of cancer cells from the lethal DNA damage induced by ionizing radiation [12–14].

 The main mechanisms through which EGFR confers radio-protection have recently been reviewed [15]. In vitro studies suggest that tumors could be sensitized to irradiation by blocking the radiation-induced nuclear import of EGFR, either through the expression of EGFR tyrosine kinase domain activating mutations or the use of cetuximab (Erbitux, Merck-Serono). Such mutations however, do not commonly occur in head and neck cancer.

 Cetuximab is an IgG1 monoclonal antibody against the ligand-binding domain of EGFR. Cetuximab binds EGFR, sequesters the receptor in the cytoplasm and eventually targets it for degradation. It has been demonstrated in vitro that this antibody enhances the radio-sensitivity in SCCHN cells [16, 17] through several processes reviewed in [18, 19].

 Because patients with locally advanced SCCHN recur locally more often than in distant sites [20, 21], it seems reasonable for patients with EGFR overexpressing tumors to receive more effective locoregional treatments. One such treatment strategy is the concomitant administration of RT with cetuximab. This rationale is supported by preclinical models, in which cetuximab acts synergistically with RT [22]. In a pivotal randomized phase III trial [23] the concomitant administration of cetuximab and RT improved locoregional control and prolonged survival compared to RT alone in patients with locally advanced SCCHN. 

 Following the introduction of cetuximab concomitantly with RT for the treatment of locally advanced SCCHN, a number of Greek oncologists used RT with concomitant administration of cetuximab and weekly cisplatin (herein named CCRT), as a treatment strategy for such patients. The background behind this approach was the fact that cetuximab increased both locoregional control and survival of such patients. Therefore, it seems logical to add cisplatin to this active combined therapeutic approach to further improve outcome, especially since this empirical approach is supported by in vitro studies [24].

 It has been shown in vitro and in tumor specimens that the expression of the ligand hepatocyte growth factor (HGF) scatter factor and its receptor HGFR (MET) increase during invasive growth of SCCHN and this pathway, by constitutively co-activating other important pathways, may play a critical role in the metastatic process of SCCHN cells [25]. 

 The ERCC1 (excision repair cross-complementation group 1), gene is one of 16 genes encoding for proteins of the nucleotide excision repair complex, which removes cisplatin-induced DNA adducts [26]. ERCC1 was shown in a randomized study [27] to be a significant predictive factor in patients with completely resected non-small-cell lung cancer (NSCLC) treated with cisplatin-based adjuvant chemotherapy. In the above study, only patients with ERCC1 negative tumors had shown benefit from the treatment. Polymorphisms in the 3′-UTR of ERCC1 and in the coding regions of the ERCC2/XPD and XRCC1 genes have been associated with disease prognosis and response to cisplatin in SCCHN patients [28].

 Matrix metalloproteinases (MMPs) are a family of zinc-dependent proteinases that play an important role in the destruction and repair of the extracellular matrix and basement membranes in various physiological and pathological processes, including gastrointestinal inflammation and carcinogenesis [29, 30]. Importantly, the activation of the MMPs liberates growth factors from the extracellualr matrix, including EGFR, FGFR and PDGFR ligands [31]. Preclinical studies have demonstrated that MMP9 plays an important role in tumor-induced angiogenesis as well, with tumor-associated inflammatory and stromal cells being the main source of the proteinase. MMP9-mediated release of vascular endothelial growth factor (VEGF) and recruitment of pericytes to the angiogenic vasculature have been postulated to be the major processes involved in MMP9-stimulated angiogenesis [32].

 In the present retrospective analysis we report our experience with the use of CCRT in patients with locally advanced SCCHN. To our knowledge this is the first report on the efficacy of this combination in such patients. Furthermore, we evaluated the association of a variety of potential tumor biomarkers with the observed response to CCRT.

## 2. Patients and Methods

### 2.1. Eligibility and Treatment

The medical records of 37 patients with newly diagnosed, histologically confirmed locally advanced nonnasopharyngeal SCCHN tumors, treated with CCRT in four centers, in which the aforementioned therapeutic strategy was adopted, were retrospectively reviewed. Patients amenable for this type of treatment had to have an age of >18 years, performance status (PS) 0-1 on the Eastern Cooperative Oncology Group (ECOG) scale and adequate bone marrow, hepatic and renal function to tolerate treatment. According to our standard practice, a written informed consent was obtained from each patient before the acquisition of biological material for research purposes.

 All patients were treated with a linear accelerator with the intention to receive definitive RT (70 Gy to the tumor area and 45 Gy to the rest of the neck) concomitantly with weekly cisplatin 40 mg/m^2^ and weekly cetuximab 400 mg/m^2^, as a loading dose during the first week and 250 mg/m^2^ on weeks 2–7. Before treatment administration, all patients were hydrated and given standard premedication. An H3-antagonist was used as antiemetic in all patients.

 Drug doses were modified according to the grade of side effects as previously described [7, 33]. Details on the RT technique, as routinely used in our centers, have been previously described as well [7]. All adverse events were graded for this analysis according to the National Cancer Institute Common Toxicity Criteria (NCI-CTC, version 3.0). The radiation Therapy Oncology Group (RTOG) criteria were used to assess RT-related toxicities.

 Approximately three months after the completion of CCRT, all patients underwent a work-up including endoscopic examination, chest x-ray, an ultrasound or computer tomography (CT) scan of the liver, and a CT scan or MRI of the head and neck region. In selected patients, especially those with a partial response (PR), an [^18^F] fluoro-deoxy-D-glucose (FDG) positron emission tomography (PET)/CT scan was also recommended. Baseline and post CCRT scans were retrospectively collected and reviewed by a radiologist (A. K-F.) experienced in head and neck topology and an independent radiologist. Response to CCRT was assessed by the RECIST criteria.

### 2.2. Tissue Microarray (TMA) Construction

Formalin-fixed paraffin-embedded (FFPE) tumor tissue from 36 patients was used for protein and gene analysis. Representative slides (H&E) from the tissue blocks were reviewed by two experienced pathologists (G. K. and M. B.) for confirmation of the diagnosis, adequacy of material and calculation of the percentage of tumor in each case. Thirty-two specimens were arrayed (2 cores per case, 1.5 mm in diameter) into a recipient paraffin block (Paraplast, McCormick, Saint Louis, MO, USA) using a manual arrayer (Beecher Instruments, Sun Prairie, WI, USA). The TMA block also included tissue cores, in the first and the last column, from skin, tonsil, placental, kidney, thyroid, ovarian, prostate, and urothelial carcinoma that served as positive and negative controls. 

### 2.3. Immunohistochemistry (IHC)

Immunohistochemical labelling was performed according to standard protocols with slight modifications [34] on serial 3 *μ*m thick sections, form the original blocks or the TMA block. As previously reported [35], the reproducibility of TMA immunostaining of different proteins compared to that obtained from whole sections of the original paraffin blocks is very high. The deparaffinization, antigen retrieval and staining procedures for EGFR [clone 31G7, Zymed (Invitrogen), Carlsbad, CA, USA; dilution 1 : 50], ERCC1 (clone 8F11, Neomarkers, Fremont, CA, USA; dilution 1:450), p16^INK4A^ (clone SPM304, Spring Bioscience, Fremont, CA, USA; dilution 1 : 150), and p-53 (clone DO-7, Dako, Glostup, Denmark; dilution 1 : 50) were performed using a Bond Max autostainer (Leica, Wetzlar, Germany). The hepatocyte growth factor receptor (HGFR/MET) protein was investigated using an antibody specific for the external domain of the beta chain of the MET protein (clone 8F11, Novocastra, Newcastle Upon Tyne, UK). After deparaffinization and antigen unmasking, the slides were incubated for 1 hour at room temperature with the MET antibody at a dilution of 1 : 50. After washing the primary antibody, the slides were incubated with a nonbiotin polymer detection system (BioGenex, San Ramon, CA) for a total of 40 minutes. The antigen–antibody complex was visualized using diaminobenzidine (BioGenex) as a chromogen. Slides were counterstained with Mayer's hematoxylin for 5 min (Leica), washed in fresh water, dehydrated, and mounted. 

 The evaluation of all IHC sections was done simultaneously by two pathologists (G. K. and M. B.) blinded as to the patients' clinical characteristics and survival data, according to previously proposed/established criteria with slight modifications. EGFR intensity of reactivity was scored using a four-tier system [36]; 0 (negative), no staining or background staining; 1+, definitive cytoplasmic staining and/or weak discontinuous membranous staining; 2+, moderate complete or incomplete membranous staining; 3+, strong complete membranous staining. Cases were considered positive when more than 10% of tumor cells showed at minimum 1+ staining, while 2+ or 3+ staining was classified as EGFR protein over-expression.

 ERCC1 evaluation of nuclear staining was done according to the criteria proposed by Olaussen et al. [27]. The above system was based on a semi-quantitative H score, which combines the stain intensity and the percentage of positive tumor cells. The median of all H scores was chosen as the cut off point for separating positive from negative cases.

 HGFR (MET) protein expression was evaluated using an intensity-adjusted scoring system (combining percentage and intensity of staining) according to Nakajima et al. [37]. Briefly, intensity scores ranged from 0 to 3 (0 = no staining, 1 = weakly positive, 2 = moderately positive, and 3 = strongly positive staining), and the staining pattern based on the percentage of positive tumor cells ranged from 0–3 (0 = 0 to 5%, 1 = 6 to 25%, 2 = 26% to 50%, and 3 = 51% to 100%). The localization of staining was either cytoplasmic or cytoplasmic/membraneous. Cases with a total score of at least 2 were considered positive (expressing tumors), whereas cases with a total score of 0-1 were grouped together and considered to be negative or low expressing tumors. Nuclear and/or cytoplasmic p16^INK4A^ staining in ≥25% of tumor cells was considered positive [38]. For p-53 protein expression, cases were scored as negative or positive, if ≤10% of nuclei or >10% of nuclei were stained, respectively [39].

### 2.4. Fluorescence in Situ Hybridization (FISH)

FISH was performed on 4.5 *μ*m thick TMA sections or whole sections of FFPE archival tissue samples using the LSI EGFR/CEP7 Dual Color Probe, (Abbott Molecular, Des Plaines, IL, USA), the LSI D7S486/CEP7 Dual Color Probe, (Abbott Molecular) and the specific for the HGFR/MET gene at region 7q31, Poseidon Repeat Free MET/SE7 probe (Kreatech Diagnostics, Amsterdam, NL). The EGFR probe, detecting a 300 kbp genomic region spanning the EGFR locus on 7p12, and the LSI D7S486 detecting a 200 kbp genomic region at region 7q31, were labelled with SpectrumOrange, while the centromere 7 specific probe (CEP7) was labelled with SpectrumGreen. The LSI D7S486/CEP7 Dual Color Probe was used to identify deletions in 7q31 that have frequently been detected in SCCHN patients, suggesting the existence of tumor suppressor genes [40]. The HGFR/MET gene probe was directly labeled with PlatinumBright550 and the SE7 (Chromosome 7 Satellite enumeration) probe with PlatinumBright495.

 FISH was performed according to the manufacturer's protocol with minor modifications. Briefly, for all probes the deparaffinized tissue sections were incubated in citric acid solution, pH 6.0 for 10 min at 98°C. After washing twice for 2 min in dH_2_O, slides were treated with a proteinase K solution for 10 min at 37°C in a hybridizer (Dako), washed for 5 min in 2xSSC solution and 1 min in dH_2_O, and dehydrated (75, 85 and 100% ethanol, each for 1 min). Five to 15 *μ*L of the probe mixture were then applied to each slide, slides were covered by cover slips, sealed with fixogum rubber cement, heat denatured for 5 min at 72°C (LSI EGFR/CEP7 and LSI D7S486/CEP7) and 80°C (MET/SE7) on a hot plate, and hybridized for at least 16 h at 37°C in a humidity chamber. After removing the cover slips by incubation in wash buffer (SSC, 0.3% NP-40), slides were washed for 7 min with wash buffer at 72°C. Subsequently, slides were dehydrated in 70%, 90% and 100% ethanol, each for 1 min, air dried protected from light, and finally nuclear counter staining was carried out with DAPI/Antifade solution (ZytoVision).

 In 3 cases, due to inadequate material for the FISH assays we perform sequential multilocus fluorescence in situ hybridization (SML-FISH) according to Walch et al., with slide modifications [41]. After image acquisition, the slides previously hybridized with the LSI D7S486/CEP7 were washed by heating the section in SSC solution at 75°C for 16 hours, followed by denaturation at 73°C for 5 minutes in 70% formamide/SSC. Then, the slides were counterstained with DAPI and examined under fluorescence (x100 oil lens) to ensure absence of fluorescent signals. The hybridization, posthybridization and nuclear counterstaining procedure for the MET/SE7 probe was performed as mentioned above.

 Slides hybridised with the EGFR/CEP7 probe were analyzed using a Zeiss fluorescence microscope (Axioskop 2 plus HBO 100) equipped with high quality objectives and an appropriate filter set. Slides hybridized with the LSI D7S486/CEP7 and MET/SE7 probes were analyzed using the Nikon 80i fluorescence microscope (Nikon GmbH, Dusseldorf, Germany) with a motorized 4 slide stage, equipped with high quality objectives (all form Nikon), an appropriate four filter set [DAPI, doublePath FIRC/TRITC, ZyGreen that is similar to Abbott Molecular SpectrumGreen and Kreatech's PlatinumBright550, and ZyOrange that is similar to Abbott Molecular SpectrumOrange and Kreatech's PlatinumBright495 (all from Chroma Technology Corp, Rockingham, VT, USA)] and an ultrasensitive black and white camera (QImaging, Surrey, BC, Canada). As a source of fluorescence illumination, the X-cite 120 (EXFO Photonic Solutions Inc, Ontario, Canada) equipped with a long-life 120-watt metal halide short arc lamp was used.

 For the assessment of the FISH assays, in the majority of the cases, over 10 fields (x100) were captured by a computer-controlled digital camera and processed by commercially available software systems (FISH Imager Metasystems, Altlussheim, Germany for EGFR/CEP7 and XCyto-Gen, Alphelys, Plaisir, France for LSI D7S486/CEP7 and MET/SE7). For the latter probes, sequential, digital images were captured by a stack motor for the DAPI (1 or 2 planes at 0.5 *μ*m), ZyGreen (5 planes at 0.85 *μ*m or 4 planes at 1.15 *μ*m) and ZyOrange (5 planes at 0.85 *μ*m or 4 planes at 1.15 *μ*m) filter settings, and the resulting images were reconstructed with blue, green and red pseudo-colors. Sixty nonoverlapping intact nuclei from the invasive part of the tumor were evaluated for each case according to morphological criteria using DAPI staining.

 The evaluation of the FISH sections was done simultaneously by two observers (G. K and M. B). For each specimen, the absolute and mean copy number per cell of each DNA probe, the total number and percentage of cells with zero, one, two, three, and >4 copies of the respective probe, homozygous and heterozygous deletions, trisomies and polysomies, as well as the gene/CEP7 ratios were calculated.

 FISH patterns for the EGFR gene were defined as previously described [42]. The status of the D7S486 locus was evaluated as follows: deletion if >35% of tumor nuclei contained one signal; trisomy/polysomy if >10% of tumor cells showed two or more copies of the D7S486 locus and chromosome 7. HGFR/MET gene status was classified according to Cappuzzo et al. [43] by six FISH strata as follows: (1) disomy (≤2 copies in >90% of the cells); (2) low trisomy (≤2 copies in ≥40% of cells, 3 copies in 10–40% of the cells, ≥4 copies in <10% of cells); (3) high trisomy (≤2 copies in ≥40% of cells, 3 copies in ≥40% of cells, ≥4 copies in <10% of cells); (4) low polysomy (≥4 copies in 10–40% of cells); (5) high polysomy (≥4 copies in ≥40% of cells); and (6) gene amplification (defined by the presence of tight EGFR gene clusters and a ratio of EGFR gene to chromosome of ≥2 or ≥15 copies of EGFR per cell in ≥10% of analyzed cells).

### 2.5. EGFR, ERCC1 and MMP9 mRNA Expression

For this retrospective study, intact RNA of high quality, as determined by analysis of the housekeeping gene RPL37A, was isolated from 33 FFPE tumour tissue samples employing an experimental method based on proprietary magnetic beads from Siemens Healthcare Diagnostics (Cologne, Germany), as previously described [44]. The number of malignant cells represented at least 30% of all nucleated cells per section, as verified by hematoxylin-eosin staining. Kinetic reverse transcription-polymerase chain reaction (kRT-PCR) was applied for the assessment of messenger RNA (mRNA) expression of EGFR, ERCC1 and MMP9 using the following TaqMan based primer/probe sets:

EGFR  Probe  CCTTGCCGCAAAGTGTGTAACGGAAT
  Forward Primer CGCAAGTGTAAGAAGTGCGAA
  Reverse Primer CGTAGCATTTATGGAGAGTGAGTCT
ERCC1 Probe TCCTCGCCTGGAGCCCCGA
  Forward Primer AGGAGCTGGCTAAGATGTGTATCCT
  Reverse Primer CCAGGTACCGCCCAGCTT
MMP9 Probe CAGGCAGCTGGCAGAGGAATACCTGTAC
  Forward Primer CCCTGGAGACCTGAGAACCA
  Reverse Primer CCACCCGAGTGTAACCATAGC

RPL37A and GAPDH were used as housekeeping (normalization) genes. Forty cycles of nucleic acid amplification were applied and the cycle threshold (CT) values of the target genes were identified. CT values were normalized by subtracting the CT value of the housekeeping gene RPL37A from the CT value of the target gene (ΔCT). RNA results were then reported as 40-ΔCT values, which correlated proportionally to the mRNA expression level of the target gene.

 Human reference total RNA pooled from ten human cell lines (Stratagene, La Jolla, CA) was used as a positive control. RNA-free DNA extracted from tumor tissues was used as a negative control.

### 2.6. ERCC1, ERCC2/XPD and XRCC1 Gene Polymorphisms

DNA from peripheral blood and FFPE tissues was normalized at 20 ng/uL. The following Taqman SNP genotyping assays were used [Applied Biosystems, Biosolutions, Athens, GR]: C_3145050_10, detecting the ERCC2 Asn312Asp (AAC/GAC) polymorphism [rs1799793]; C_3145033_10, detecting the ERCC2 Lys751Gln (AAG/CAG) polymorphism [rs13181]); C_622564_10, detecting the XRCC1 Gln399Arg (CAG/CGG) polymorphism [rs25487]; and C_2532948_10, detecting the ERCC1 C8092A/CD3EAP Q504K (Gln/Lys) polymorphism [rs3212986]. Of note, the sequence detected by this assay (CACAGGCCGGGACAAGAAGCGGAAG[C/A]AGCAGCAGCAGCAGCCTGTGTAGTC), which matches previous reports [45], includes a polymorphism in the 3′-UTR of the ERCC1 gene, which is simultaneously located at the 3′end of the CD3EAP coding region (http://www.ncbi.nlm.nih.gov/SNP/snp_ref.cgi?rs=3212986), since these genes are located in opposite directions at 19q13.3. Thus, CTG>CTT (G/T change) is the forward sequence in ERCC1, corresponding to the reverse CAG>AAG (C/A change) in CD3EAP. Runs were performed in duplicates in 10 *μ*L reactions with 40 ng DNA input, amplified for 40 cycles under standard conditions in an ABI7500 real time PCR system equipped with the SDS v1.4 software keeping the default parameters (Applied Biosystems, Biosolutions, Athens, GR). Negative control did not provide amplification curves, while sample amplification curves were considered for further analysis if the cycle threshold (Ct) for the detected products was <38. Differences of the mean Cts (dCt) for the two alleles detected by each assay were: −1.93 for ERCC1 C8092A, 0.47 for ERCC2 N312D, 1.55 for ERCC2 K751Q, and 1.34 for XRCC1 Q399R, all within the acceptable limits for this type of assays (±2) (Applied Biosystems).

### 2.7. EGFR and kRAS Mutation Analysis

Genomic DNA was derived from FFPE tumors as previously described [45]. Samples consisting of >75% tumor cells were considered as eligible for DNA extraction and sequence analysis, otherwise macrodissection was performed to increase the tumor cell content to >75%.

 We amplified exons 18, 19, 20, and 21 of the EGFR tyrosine kinase domain from genomic DNA (primary tumor tissue DNA) and germline DNA (peripheral blood DNA) that was extracted with the Invisorb Spin Blood Midi Kit (Invitek GmbH, Berlin, Germany) according to the manufacturers instructions. All PCR's were conducted as previously described [46]. All mutations were reconfirmed by PCR amplification and analysis of an independent DNA isolate. Exons 18, 19, 20, and 21 were reconfirmed in all patients identified as harboring mutations. Germline DNA was analyzed on two separate occasions for exons 18, 19, 20, and 21 for all patients with mutations, in order to confirm EGFR mutations as somatic or germline in origin. kRAS mutation analysis of codons 12 and 13 was performed as previously described [47].

 All PCR products were purified by solid-phase reversible immobilization chemistry followed by bi-directional dye-terminator fluorescent sequencing. All exons were sequenced with the inner forward and reverse primers used for PCR. Sequences were analyzed by BLAST and chromatograms by manual review, and compared to: EGFR mRNA reference sequence Accession number NM 005228 and/or the EGFR gene sequence Accession number AF288738; RAS mRNA GI 34485723 and/or the RAS gene sequence GI 14277199 (http://www.ncbi.nlm.nci/).

 The EGFR exon 21 mutation, L858R, was also analyzed by PCR/RFLP based on the presence of a new *Sau96I* restriction site created by the mutation. Deletions in exon 19 were also analyzed for using high performance gel electrophoresis (>2.5% agarose).

### 2.8. HPV Detection

Detection of HPV-16 and HPV-18 DNA was performed by one of the authors (A. L.) and was based on amplification of the E6 region as adopted from Ogura et al. [48], with minor modifications. Briefly, each reaction contained 0.2–0.4 *μ*g DNA template in 10 mM Tris, pH 8.3, 50 mM KCl, 1.5 mM MgCl_2_, 200 mM dNTPs, 1.5 units *Taq* DNA polymerase (Fermentas), and 100 pM of each of the primers in a total volume of 50 *μ*L. Sense and antisense primer sequences for HPV-16 E6 were 5′-AAGGGCGTAACCGAAATCGGT-3′ and 5′-GTTTGCAGCTCTGTGCATA-3′, respectively. The same sense primer was used for HPV-18 E6. The antisense primer sequence for HPV-18 E6 was 5′-GTGTTCAGTTCCGTGCACA-3′.

 The reaction mixure was subjected to PCR amplification using the GeneAmp PCR system 9700 thermal cycler (ABI). PCR cycling conditions consisted of 7 min at 96°C and 1 min at 72°C, followed by 35 cycles, including a denaturation step at 94°C for 30 s, an annealing step at 55°C for 30 s and an elongation step at 72°C for 45 s. The final extension step was carried out at 72°C. To avoid false positive and/or negative results a control (no template DNA) and an HPV positive DNA sample were included.

### 2.9. Statistical Analysis

Data on selected patient or tumor characteristics, and acute toxicity were obtained from the records. Responses were summarized as number of patients and corresponding percentages. Comparisons of the number of responders according to biomarkers were performed using the Fisher's exact test.

 Overall survival (OS) was measured from treatment initiation to patient's death or last contact. Progression-free survival (PFS) was measured from treatment initiation to verified disease progression, death or last contact. In the analysis of PFS, death without prior verified progression was encountered as event. OS and PFS were estimated by the Kaplan-Meier method. For all comparisons, level of significance was set at *a* = 0.05.

## 3. Results

### 3.1. Patients' Compliance and Toxicity

Totally, 37 patients fulfilling the eligibility criteria were included in this retrospective analysis. There were 27 men and 10 women with a median age of 60 years (Table 1). Thirty-five patients (95%) completed CCRT. One patient discontinued CCRT after the completion of the 6th week of treatment due to grade 3 thrombocytopenia. One patient, a 74-year-old man, with a history of angina and atrial fibrillation died from acute myocardial infarction during the second week of RT. In the process of reviewing the clinical data, 5 more patients were identified to have had fatal events during the 3-month period post CCRT. More analytically, one of the patients from progressive disease, while a second patient, a 60-year-old man with an unremarkable medical history, from cardiac arrest, one week after the completion of CCRT. Autopsy was refused by his relatives. A third patient, a 46-year-old man, died from massive haemorrhage of the upper aerodigestive truck, 11 weeks post CCRT. Autopsy suggested that the fatal event was attributed to bleeding from a mucosal ulceration on the right pyriform sinus. No evidence of tumor was found. The latter patient, even though a post CCRT scan was not performed, was considered in the present analysis to be complete responder. The fourth patient, a 60-year-old man, alcoholic and heavy smoker, was at the initiation of CCRT on treatment for pulmonary tuberculosis with isoniazid and rifambicin. He died 7 weeks post CCRT. Further medical information about the cause of death could not be obtained. The fifth patient, a 56-year old man with alcoholic cirrhosis died 12 weeks post CCRT from massive bleeding due to the rupture of esopharyngeal varices. The above patients were included in the analysis for response on an “intent to treat” basis. Severe side effects most commonly noticed were leukopenia (70%), dysphagia (62%), skin rash (65%), and anemia (51%) (Table 2).

### 3.2. Response to CCRT and Survival

**Figure 1:**
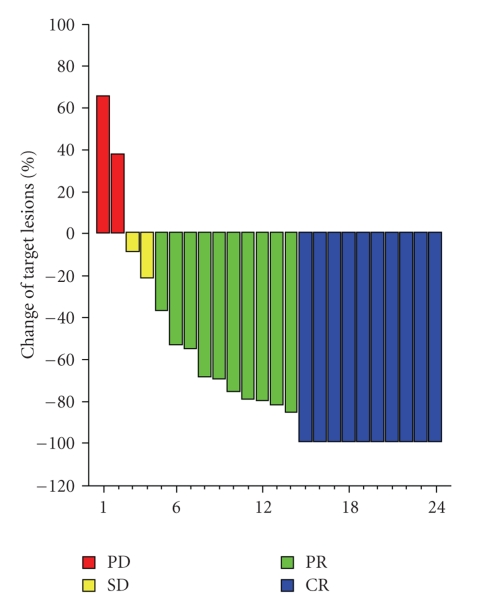
Waterfall for the response of target lesions according to RECIST criteria (*N* = 24).

Following the completion of CCRT, response was evaluated according to the RECIST criteria for 24 out of 37 patients (Figure 1). For 6 of these patients response was evaluated by PET as well. For one of the patients, response was classified as partial by RECIST, while PET was free of tumor, thus this patient was considered to be a complete responder in the overall response evaluation. In one additional case, response was evaluated by PET only.

 Of the remaining 12 non-evaluable patients, one did not have a CT examination, for 5 patients the CT examinations were not available for central review, while 6 patients died before their response evaluation. However, for one of the latter patients an autopsy was performed and no evidence of tumor was found. This patient is considered to be a complete responder.

 Overall, 11 patients (30%, 95% CI 16%–47%) achieved a CR and 11 (30%, 95% CI 16%–47%) a PR. Stable disease was seen in 3 patients (8%, 95% CI 2%–22%) and progressive disease in 5 patients (14%, 95% CI 5%–29%). For one patient the CT examination was not available, and therefore was not evaluated for response. Notably, among three patients with radiological PR that underwent an FDG-PET/CT, one of them had a negative examination. Therefore, this patient was considered as a complete responder in the final analysis. Taking into account the one patient with no evidence of tumor in the autopsy, 13 patients were considered as having achieved a CR (35%, 95% CI 8%–52%) and 10 as having achieved a PR (27%, 95% CI 14%–44%). 

 After a median follow-up of 21.3 months, 15 patients had a PFS event (10 patients demonstrated disease progression and 5 died of other causes), while a total of 9 patients had died. One-year progression-free and overall survival was 63% and 80%, respectively.

### 3.3. Immunochemistry and FISH

**Figure 2:**
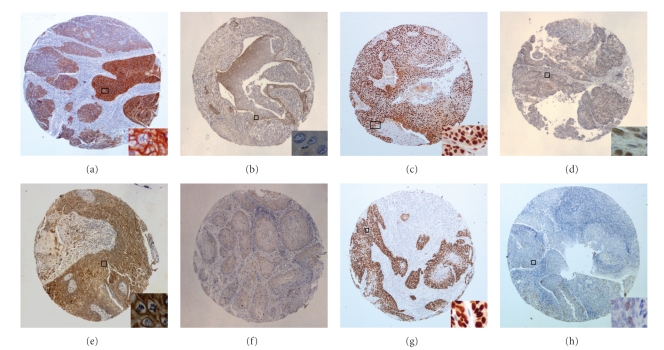
Immunohistochemistry performed on tissue microarrays. (a) EGFR protein expression in all tumor cells with focal intense complete membranous staining (+3); (b) EGFR negative case showing mild cytoplasmic focal staining; (c) ERCC1 protein strong nuclear positivity; (d) ERCC1 protein expression with equal intensity in neoplastic cells and stromal fibroblasts (regarded as negative staining); (e) MET strong cytoplasmic and membraneous protein expression; (f) Lack of MET protein expression in tumor cells; (g) p-53 strong nuclear protein expression; (h) p-53 expression in a small fraction of tumor cells (regarded as negative staining). Original magnification x20; insets (a), (c), and (g) x200; insets (b), (d), (e), and (h) x400.

**Figure 3:**
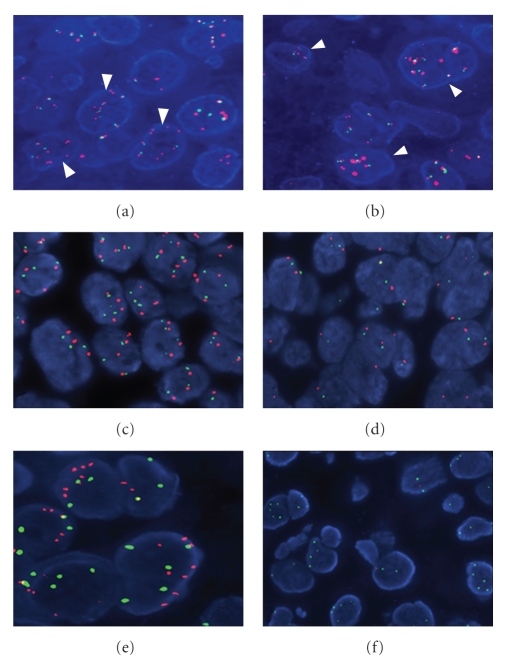
Fluorescence in situ hybridization with gene and centromeric specific probes. (a) and (b) Neoplastic nuclei showing polysomy of chromosome 7 (CEP7, green signals) and EGFR high level gene gain (red signals, arrowheads); (c) Neoplastic nuclei showing trisomy or polysomy of the MET gene (red signals) and SE7 (green signals); (d) Representative area from a case without genetic alterations. The majority of the neoplastic nuclei have 2 copies of the MET gene and SE7; (e) High polysomy of the D7S486 locus (red signals); (f) Deletion of the D7S486 gene locus in tumor cells, as defined by the presence of a single gene locus probe signal (red signals) and two CEP7 signals (green signals), or by the simultaneous lack of both of the gene locus signals and the presence of CEP7 signals (hemizygous and homozygous deletion, resp.).

Individual EGFR, ERCC1, MET, p16^INK4A^, and p-53 IHC and FISH data along with selected patient characteristics and responses are presented in Tables 3 and 4. In summary, thirty-one of 32 tumor samples (97%) were found to be EGFR positive, while in 22 samples (69%) EGFR was overexpressed (Figures 2(a) and 2(b)). No association between EGFR overexpression and complete response was identified (9/22 CRs among patients with EGFR overexpression versus 2/10 CRs among patients without EGFR overexpression; *P* = .425). One sample was EGFR amplified (Figures 3(a) and 3(b)).

 The ERCC1 protein was expressed (Figures 2(c) and 2(d)) in 27 out of 33 tumor samples (82%). No association between ERCC1 expression and response was found (9/27 responders among ERCC1 positive patients versus 2/6 responders among ERCC1 negative patients; *P* = .999).

 The MET protein was expressed (Figures 2(e) and 2(f)) in 14 out of 33 tumor samples (42%). The MET protein was detected as membraneous discontinuous or complete staining and/or cytoplasmic staining. In a small number of cases the endothelial cells of stromal vessels showed mild to moderate staining. No association between MET protein expression and complete response was found (4/14 complete responders among MET positive patients versus 7/19 complete responders among MET negative patients; *P* = .719). However, when considering objective response (CR or PR), a significant association was identified with MET protein expression (5/14 responders among MET positive patients and 15/19 responders among MET negative patients; *P* = .029).

MET gene gain was observed in 23 of 31 cases (74%). More specifically, low trisomy was detected in 16 cases, low polysomy in 6 cases, while high polysomy was identified in 1 case (Figures 3(c) and 3(d)). MET gene status was not found to be associated with response (2 responders among 8 patients with normal MET gene status versus 9 responders among 23 patients with MET gene gain, *P* = .676).

 The p16^INK4A^ protein was detected in 8 out of 30 cases examined (27%). In addition, in 5 of the 22 negative cases, p16 was highly expressed in the dysplastic squamous epithelium. Two of them showed p16 expression mainly in the dysplastic epithelium and to a small degree in scattered infiltrative neoplastic cells. No association was found between p16 and HPV-16 (*P* = .290). Furthermore, p16 was not found to be associated with response (4 responders among 8 patients with positive p16 status versus 7 responders among 22 patients with negative p16 status, *P* = .417).

 The p-53 protein was found to be expressed (Figures 2(g) and 2(h)) in 22 of 33 patients (67%). No significant association with complete response was identified (6/22 complete responders among p-53 positive patients versus 5/11 complete responders among p-53 negative patients; *P* = .437).

 Moreover, no significant association between the status of the D7S486 locus (Figures 3(e) and 3(f)) and response was identified.

### 3.4. EGFR, ERCC1, and MMP9 mRNA Expression

Individual EGFR, ERCC1 and MMP9 mRNA data along with selected patient characteristics and responses are presented in Tables 3 and 4. For all three genes the median was used as a pre-defined cut-off in order to classify tumors with high (above the median) or low (below the median) mRNA expression. The median normalized EGFR mRNA expression was 34.9 (29.6–39.5). High EGFR mRNA expression, was not found to be associated with complete response (4/17 complete responders among patients with low EGFR mRNA expression, versus 8/16 complete responders among patients with high EGFR mRNA expression; *P* = .157).

 Similarly, the median normalized ERCC1 mRNA expression was 34.8 (30.0–39.5), while no association between high ERCC1 mRNA expression and complete response was identified. Specifically, in the group of 17 patients with low ERCC1 mRNA expression 4 patients achieved a complete response, versus 8 complete responders among the 16 patients with high ERCC1 mRNA expression (*P* = .157).

Finally, the median normalized MMP9 mRNA expression was 34.3 (29.5–39.5). Only 4 of the 17 patients with low MMP9 mRNA expression achieved a complete response, while 8 of the 16 patients with high MMP9 mRNA expression demonstrated a complete response to treatment (*P* = .157). Although MMP9 mRNA expression was not found to be significantly associated with complete response, a significant association with the objective response (CR or PR) was identified (6/17 responders among patients with low MMP9 mRNA expression versus 14/16 responders among patients with high MMP9 mRNA expression, *P* = .004).

### 3.5. ERCC1, ERCC2/XPD, and XRCC1 Gene Polymorphisms

Samples from 36 patients were considered for allelotyping, including 10 from tumor tissue only, 3 from peripheral blood (germline) only and 23 from matched peripheral blood and tumor tissue. The incidence of allelic combinations in germline and tumor tissues is shown in Table 5, while individual data on ERCC1, ERCC2 and XRCC1 gene polymorphisms are presented in Table 4. Briefly, heterozygous polymorphic alleles were common for all targets; concerning homozygous combinations, C8092C was the most frequent genotype for ERCC1, Asp312Asp and Lys751Lys for ERCC2/XPD and Arg399Arg for XRCC1. In 2/10 unmatched tumor tissue samples, allelotyping data could be obtained for ERCC1 but not for ERCC2 and XRCC1, probably due to poor FFPE DNA quality. Overall, the incidence of allelic variants observed in the present study was in accordance with relevant previous data [28].

 The germline genotype did not always match the tumor genotype in the same patient, as deduced from the high dCts (5.8, 7.3, and 7.9 in three cases) in the respective tumor samples or from the amplification of allele targets that were negative in the matching peripheral blood samples. Changes in tumor genotypes were observed upon repeated testing in 4/23 patients (17%) with matched peripheral blood and tumor samples available for comparison (Table 4). Germline heterozygocity was replaced in one case by homozygocity for the rare A/A allele for ERCC1 C8092A/CD3EAP Q504K, indicating a Lys/Lys genotype for CD3EAP in the tumor. In two additional cases, germline A/G was replaced by G/G for ERCC2-312 (change of Asn/Asp into Asp/Asp in the tumor). In another case, germline ERCC2-312 Asp/Asp (no amplification of the Asn target) was replaced by Asn/Asp (dCt = 1.1) in the matched tumor tissue.

### 3.6. Mutational Analysis

Only one patient had a somatic EGFR mutation on exon 20, a D770insGF insertion. No patients were found with a kRAS codon 12/13 mutation. Additionally, no patients were identified with an L858R EGFR mutation or codon 19 deletion by alternative methods.

### 3.7. HPV Detection

We examined the presence of HPV-16 and 18 E6 in 30 patients by PCR. Totally, 6 out of 30 samples (20%) tested were HPV-16 positive (one laryngeal, 3 oral cavity and 2 oropharyngeal tumors). All samples proved to be HPV-18 negative. Interestingly, 4 of the 6 HPV-16 positive patients, who were evaluable for response, achieved a CR post CCRT.

## 4. Discussion

The present report describes our collective experience with CCRT in patients with locally advanced SCCHN. The CR rate achieved in such a heterogeneous group of patients was 35%. Additionally, 10 patients (27%) were considered as having a PR. Interestingly, one patient with a PR had a negative FDG-PET/CT after the completion of CCRT and was considered as having a CR. It is well known that assessment of response to chemo-radiotherapy in patients with SCCHN is not accurate, since a number of them are considered by radiologists as having partial response, because of residual abnormalities in posttreatment CT scans. During the last few years FDG PET/CT scans had been increasingly used for initial staging and assessment of tumor response in SCCHN [49]. Several investigators have shown that FDG-PET/CT can more accurately predict the lack of residual disease both at the primary site and the neck (negative predictive value 100%, sensitivity 100% and specificity 96%) [50, 51] and it has therefore been considered to be a valuable clinical tool in the management of SCCHN.

 The review of our clinical data showed that the treatment was feasible and that the compliance of the patients was satisfactory, since all except two completed RT. It is well known that most patients with SCCHN belong to low social-economic status, are alcoholic, heavy smokers, and bear serious co-morbidities. Furthermore, serious toxic sequelae of chemo-radiotherapy, such as dehydration, infections, malnutrition, and excessive weight loss may deteriorate their general heath status and contribute to fatal events. A high incidence of unexpected severe adverse events, including fatal events, was described by Pfister et al. [52] in a phase II study and was confirmed in our retrospective analysis of an unselected SCCHN population. These patients should therefore be closely monitored during CCRT and the immediate period following CCRT.

 The discovery of predictive factors in treatments, such as RT concomitantly with cetuximab, is of paramount importance, since this regimen is emerging as the new standard for patients with SCCHN. Unfortunately, to date the identification of such molecular predictors remains elusive. In the present analysis, we evaluated potential associations of EGFR, MET, ERCC1, and MMP9 with response to CCRT. 

 Even though high EGFR protein expression has been reported to be predictive for increased tumor response in patients with SCCHN treated with conventional fractionated [9] or accelerated [53, 54] RT, this finding has not been confirmed in randomized studies in patients with recurrent and/or metastatic SCCHN treated with gefitinib [55] or cisplatin and cetuximab [56]. Contrary to what would be expected, patients with low to moderate EGFR protein expression demonstrated a higher response rate to the combination of cisplatin and cetuximab than those with high EGFR expression. In our retrospective analysis, we were not able to find a correlation between EGFR protein expression and response to CCRT. 

 We have also assessed EGFR gene copy number by FISH. We found that in most of the tumors EGFR polysomy but not amplification was evident; however, it was not correlated with response. These findings are in agreement with other trials, showing that the prevalence of EGFR amplification in SCCHN is low [57, 58] and that EGFR gene copy numbers are not correlated with tumor response in patients with recurrent/metastatic SCCHN, who nevertheless responded to the EGFR tyrosine kinase inhibitors (TKIs) erlotinib or gefitinib [59, 60]. It has been reported that in nonsmall cell lung cancer (NSCLC), mutations within the EGFR tyrosine kinase domain, mainly in exons 18, 19 and 21, confer sensitivity to TKIs [61]. However, such mutations are rare in SCCHN, ranging from 1% to 7% in caucasian/white and asian patients, respectively [60, 62, 63]. In a study of 134 SCCHN tumors, direct DNA sequencing could not identify any mutations [58]. In line with these findings, we screened 31 tumors for EGFR mutation in exons 18, 19 and 21 and were able to identify only one patient harboring an EGFR mutation. Apparently, due to the very low prevalence, EGFR mutations cannot be used as predictors of response to anti-EGFR treatment in SCCHN. 

 Clearly, further studies are needed to fully elucidate the mechanisms of sensitivity and resistance to cetuximab or EGFR TKIs. It is possible that other factors that are further downstream in the EGFR pathway and/or the interplay of the EGFR pathway with other activated pathways are more important than EGFR alone in modulating responses to anti-EGFR treatments. 

 Additionally, we assessed MET protein expression by IHC and gene copy number by FISH. To our knowledge, this is the first study attempting to correlate MET with response to concomitant RT with cisplatin and cetuximab. MET protein expression was noted in 14 of 33 of tumors studied and the gene was amplified in 5 of the patients. It appears that, as in the case of NSCLC [64, 65], MET gene amplification is an infrequent event in SCCHN as well and is not associated with responses to CCRT.

 Interestingly, the present retrospective analysis is one of a few studies that have investigated a potential association between ERCC1 protein expression and response to CCRT in patients with SCCHN. It is noteworthy, that knowledge regarding the role of ERCC1 in SCCHN is very limited. Recently, Handra-Luca et al. [66] reported that low ERCC1 protein expression was associated with higher rates of tumor response (79% versus 56%, *P* = .04) and lower risk of cancer-specific death (risk ratio 0.42, *P* = .04) in patients with SCCHN treated with cisplatin-based induction chemotherapy. However, this positive association was not confirmed in a similar study recently conducted by our group [67] and in the present analysis. The reasons for this discrepancy, regarding the predictive role of ERCC1, are not clear. Small sample size, differences in the treatment regimens, lack of standardization of the IHC methodology for assessing ERCC1 protein expression, and differences in patient characteristics, stage and tumor location maybe a few, but certainly not the only factors responsible for the conflicting results.

 An important finding of the present retrospective analysis was that high MMP9 mRNA expression, assessed by kRT-PCR, was significantly associated with objective response. Positive correlations have been observed between MMP9 mRNA expression levels and metastatic spread of SCCHN tumors [68]. Overexpression by MMP9 may in part be regulated via nuclear factor kappa B (NF-kB) [69]. In addition, inflammatory processes induced by HPV infections could activate MMPs, which would in turn liberate EGFR ligands from the extracellular matrix, thereby promoting HNSCC tumor progression through increased EGFR signalling. It appears therefore, that MMP9 positive tumors could be particularly sensitive to EGFR inhibition. This notion is in complete agreement with our finding that high MMP9 mRNA expression is significantly associated with objective response to cetuximab containing chemotherapy. However, further analysis is needed in noncetuximab treated SCCHN patients, to evaluate whether MMP9 might be a “poor prognosis marker” turned onto an “improved response marker” by the addition of cetuximab to RT or CCRT.

 Regarding four commonly studied polymorphic sites in ERCC1, ERCC2/XPD, and XRCC1, it was interesting to identify discordant tumor tissue/peripheral blood genotypes. This may be worthy considering when assessing polymorphisms as prognostic/predictive markers in oncology, since most such available data, including polymorphisms in excision repair genes, derive from determinations in peripheral blood (germline) DNA [28, 45, 70]. As indicated by the diminished presence of one allele with real time PCR, discordant genotypes in 3 out of 4 cases might correspond to loss of heterozygosity (LOH) of the corresponding alleles in the tumor. LOH can be inferred upon SNP-genotyping [71]. This finding needs further validation, while its biological impact, if any, is presently unknown, since LOH in ERCC1 and ERCC2/XPD has not been studied in SCCHN. LOH does not seem to be a common or major event in colorectal carcinogenesis [72]. Nevertheless, other than previously reported [28], we did not observe an association between tumor excision repair gene polymorphisms and patient outcome, possibly due to the small sample size, while the investigated polymorphism in ERCC1 was not related to the corresponding mRNA and protein expression. 

 Importantly this is the first report on the sensitivity of HPV-associated SCCHN to cetuximab-containing CCRT. There is a large body of molecular evidence suggesting that HPV (mainly HPV-16 and HPV-18) plays an important role in the pathogenesis of SCCHN and particularly of oropharyngeal tumors [73, 74]. HPV-16 is the most prevalent genotype in SCCHN, accounting for more than 90% of positive cases [75]. We assessed the presence of HPV by PCR, since this detection method is probably more sensitive than other methods, such as in situ hybridization [76]. The frequency of the presence of HPV, predominantly the HPV-16 genotype, in Greek patients with oropharyngeal or laryngeal cancer was 43% and 40%, respectively [77, 78]. Finally there are several lines of evidence suggesting that, HPV-associated SCCHN has a better prognosis than SCCHN in HPV-negative patients, possibly due to enhanced radio-sensitivity or the absence of field cancerization [79]. These data are in complete accordance with the findings of our study, in which exclusively HPV-16 DNA was detected in 6 (20%) of our patients. Notably, 4 of these patients were evaluable for response and all of them demonstrated a CR after the completion of CCRT. The observed high responsiveness of the HPV-positive patients might possibly be due to activation of MMP9. All 4 of the above patients exhibiting a CR had high MMP9 mRNA expression. Activation of MMP9 could liberate EGFR ligands from the extracellular matrix, thereby promoting HNSCC tumor progression through increased EGFR signalling. MMP9 positive tumors could therefore be, as discussed earlier, particularly sensitive to EGFR inhibition with cetuximab.

 The p16 overexpression reported here was not associated with presence of HPV-16, in contrast to previous studies [80, 81]. As previously shown, p16 overexpression is not limited to HPV-16 positive cases [82], since a small number of cases with HPV negative genotype showed very high p16 expression. Furthermore, the finding of p16 overexpression in HPV-16 negative tumors may be the result of oncogene-driven cellular senescence or infection with other viruses that down-regulate retinoblastoma protein expression [38]. The above combined with the small number of positive cases could explain the lack of association between p16 and HPV-16 positivity reported in our study. However, other contributing factors, such as differences in antibody specificity and limitations of the immunohistochemical and PCR assays cannot be excluded.

 As shown in patients with NSCLC, in colorectal or pancreatic cancer patients treated with anti-EGFR targeted treatments reviewed in [83], there is a subgroup of patients that is particularly benefited from such treatments, that is, those who develop the typical acne-like or maculopapular rash [57]. In the present analysis, rash of any grade was not found to be associated with response to CCRT. Likewise, lack of a correlation between the development of rash and response to cetuximab was reported in two other studies in patients with recurrent/metastatic SCCHN [84, 85]. However, in both of these studies, rash was a predictor for survival and in one of them [84] for time to progression (TTP), as well. Whether rash will be found to be significantly correlated with TTP or survival remains to be seen with longer followup. Notably, none of our patients discontinued CCRT due to severe RT-induced dermatitis, which has occasionally been reported in patients with SCCHN treated with RT concomitantly with cetuximab [86]. Nevertheless, intensive medical treatment should be offered to these patients by experienced dermatologists, since in several cases there is a considerable risk for secondary skin infections.

 In conclusion, it appears from the present retrospective analysis that, CCRT is feasible in patients with locally advanced SCCHN. However, extremely close monitoring is required for patients with serious co-morbidities, during CCRT and the 3-month posttreatment period, because such patients are at high-risk for dying from nontreatment related causes. The status of all the genes evaluated in this analysis, except MMP9, was not of predictive value to CCRT. High MMP9 mRNA expression, assessed by kRT-PCR, was found to be significantly associated with objective response. It appears that MMP9 might be of predictive value in SCCHN patients treated with cetuximab. However, it has to be kept in mind that, given the retrospective nature of the present analysis and the relatively small number patients, a selection bias cannot be excluded. Therefore, our findings should by no means be considered as definitive, but rather as hypothesis generating for future prospective trials.

## Figures and Tables

**Table 1:** Patient characteristics (*N* = 37).

Age		
Median	59	
Range	36–82	
	*N*	%
Gender		
Men	27	73
Women	10	27
Performance status		
0	33	89
1	3	8
2	1	3
Primary site		
Oral cavity	12	32
Larynx	11	30
Oropharynx	8	22
Hypopharynx	4	11
Paranasal Sinuses	1	3
Major Salivary Glands	1	3
Stage		
II	2	5
III	6	16
IV	29	78

**Table 2:** Worst toxicity expressed as *N* (%) during CCRT (RTOG criteria).

	Grade 1	Grade 2	Grade 3	Grade 4
Anemia	12 (32)	6 (16)	1 (3)	0 (0)
Neutropenia	3 (8)	13 (35)	7 (19)	0 (0)
Leucopenia	4 (11)	11 (30)	11 (30)	0 (0)
Thrombocytopenia	4 (11)	3 (8)	2 (5)	0 (0)
Nausea/vomiting	11 (30)	5 (14)	1 (3)	0 (0)
Fatigue	5 (14)	9 (24)	3 (8)	0 (0)
Dysphagia/anorexia	2 (5)	14 (38)	7 (19)	0 (0)
Weight Loss	2 (5)	2 (5)	1 (3)	0 (0)
Dermatitis	3 (8)	7 (19)	2 (5)	0 (0)
Rash	8 (22)	12 (32)	3 (8)	1 (3)
Mucositis	9 (24)	9 (24)	5 (14)	0 (0)
Mouth dryness	6 (16)	7 (19)	0 (0)	0 (0)
Constipation	10 (27)	1 (3)	1 (3)	0 (0)
Diarrhea	0 (0)	1 (3)	1 (3)	0 (0)
Infection	2 (5)	0 (0)	1 (3)	0 (0)
HSR	4 (11)	2 (5)	0 (0)	0 (0)
Otitis	0 (0)	3 (8)	0 (0)	0 (0)
Hoarseness	3 (8)	4 (11)	1 (3)	0 (0)
Peripheral Neuropathy	0 (0)	1 (3)	0 (0)	0 (0)
Nephrotoxicity	0 (0)	1 (3)	0 (0)	0 (0)
Confusion	1 (3)	0 (0)	0 (0)	0 (0)
Dizziness	3 (8)	0 (0)	0 (0)	0 (0)
Pruritus	2 (5)	0 (0)	0 (0)	0 (0)
Bleeding	3 (8)	0 (0)	0 (0)	0 (0)
Pain	1 (3)	1 (3)	0 (0)	0 (0)
Dry skin	1 (3)	0 (0)	0 (0)	0 (0)
Memory loss	1 (3)	0 (0)	0 (0)	0 (0)
Seizure	1 (3)	0 (0)	0 (0)	0 (0)

**Table 3:** Selected patient and tumor characteristics, EGFR, MET, p-53, HPV-16, and p16 status and response to CCRT.

n	Primary site	Gender	Age	Response	EGFR	EGFR	EGFR	MET	MET	MET	p-53	HPV-16	p16
			(years)		(IHC)	(FISH)	(mRNA)	(IHC)	(FISH)	(FISH)	(IHC)	(DNA)	(IHC)
(1)	Oral cavity	W	69	PR	2+	LLG	H	N	TR	GAIN	5	N	N
(2)	Oral cavity	M	66	PR	2+	DI	H	P	TR	GAIN	>90	N	N
(3)	Oral cavity	M	59	PR	2+	TR	H	N	TR	GAIN	>90	N	N
(4)	Oral cavity	M	82	CR	3+	DI	H	N	LP	GAIN	>90	P	N
(5)	Oral cavity	M	61	NE	3+	TR	L	P	LP	GAIN	>90	P	N
(6)	Oral cavity	M	69	PD	1+	DI	L	N	DI	NORMAL	>90	—	—
(7)	Oral cavity	W	41	PD	3+	DI	L	P	TR	GAIN	80	—	N
(8)	Oral cavity	W	60	CR	3+	TR	H	P	TR	GAIN	0	N	N
(9)	Oral cavity	M	44	CR	3+	TR	H	P	TR	GAIN	30–40	P	P
(10)	Oral cavity	M	60	ED	2+	DI	L	P	TR	GAIN	>90	N	P
(11)	Oral cavity	W	59	PD	3+	DI	H	P	LP	GAIN	20–30	N	N
(12)	Oral cavity	W	55	CR	—	—	Undet.	—	—	—	—	—	—
(13)	Oropharynx	M	57	CR	3+	AMPL	H	P	LP	GAIN	>90	N	N
(14)	Oropharynx	W	36	CR	2+	DI	L	N	LP	GAIN	<5	P	P
(15)	Oropharynx	M	59	CR	3+	DI	L	N	LP	GAIN	0	N	N
(16)	Oropharynx	W	55	CR	3+	DI	L	P	LP	GAIN	30–40	P	P
(17)	Oropharynx	M	69	PR	3+	DI	Undet.	N	DI	NORMAL	—	—	P
(18)	Oropharynx	M	46	CR	NE	—	H	—	—	—	—	N	P
(19)	Oropharynx	M	73	PD	1+	TR	H	N	TR	GAIN	0	N	N
(20)	Oropharynx	M	67	SD	3+	TR	H	P	DI	NORMAL	70–80	N	N
(21)	Hypopharynx	M	46	ED	3+	TR	H	N	HP	GAIN	>90	N	—
(22)	Hypopharynx	W	64	PR	NE	—	L		—	—	—	N	—
(23)	Hypopharynx	M	56	ED	3+	TR	L	P	DI	NORMAL	>90	P	N
(24)	Hypopharynx	W	56	PR	3+	DI	L	N	—	—	0	—	—
(25)	Larynx	M	55	CR	3+	DI	H	N	DI	NORMAL	0	N	N
(26)	Larynx	M	68	PR	2+	DI	L	N	TR	GAIN	>90	N	N
(27)	Larynx	M	60	ED	—	—	Undet.	—	—	—	—	—	—
(28)	Larynx	M	42	PR	3+	DI	L	N	DI	NORMAL	>90	N	N
(29)	Larynx	M	76	CR	3+	LLG	L	N	DI	NORMAL	30–40	N	N
(30)	Larynx	M	74	ED	NE	DI	L	N	—	—	0	N	—
(31)	Larynx	W	46	SD	3+	DI	L	P	TR	GAIN	5–10	N	P
(32)	Larynx	M	74	ED	2+	DI	L	P	TR	GAIN	>90	N	P
(33)	Larynx	M	67	PD	3+	LLG	H	N	TR	GAIN	>90	N	N
(34)	Larynx	M	54	CR	2+	DI	H	N	TR	GAIN	0	N	N
(35)	Larynx	M	54	PR	3+	DI	Undet.	N	DI	NORMAL	—	—	N
(36)	Paranasal Sinuses	M	65	SD	3+	LLG	H	P	TR	GAIN	>90	N	N
(37)	Major Salivary Gland	M	74	PR	3+	TR	L	N	LP	GAIN	>90	N	N

n = sample order number, M = man, W = woman.

CR = complete response, PR = partial response, SD = stable disease, PD = progressive disease, NE = nonevaluable, ED = early death.

LLG = low level gain, DI = disomy, HP = high polysomy, LP=low polysomy, TR = trisomy, AMPL = amplification.

P = positive, N = negative, H = high, L = low, Undet. = undetermined by real time PCR.

**Table 4:** Selected patient and tumor characteristics and response to CCRT in comparison to excision repair genes and MMP9 status.

					ERCC1	ERCC1	ERCC1 C8092A/	ERCC2-	ERCC2-	XRCC1-	MMP9
n	Primary site	Gender	Age	Response	(IHC)	(mRNA)	CD3EAP Q504K#	312#	751#	399#	(mRNA)
(1)	Oral Cavity	W	69	PR	N	H	A/A	Asn/Asp	Gln/Lys	Arg/Arg	H
(2)	Oral Cavity	M	66	PR	P	H	C/C	Asp/Asp	Lys/Lys	Arg/Arg	H
(3)	Oral Cavity	M	59	PR	P	L	C/C	Asp/Asp	Lys/Lys	Arg/Arg	H
(4)	Oral Cavity	M	82	CR	P	H	C/C	Asp/Asp	Lys/Lys	Gln/Arg	H
(5)	Oral Cavity	M	61	NE	P	L	A/C	Asp/Asp^⋀^	Gln/Lys	Gln/Arg	L
(6)	Oral Cavity	M	69	PD	P	L	A/C*	Asn/Asn*	Gln/Gln*	Gln/Gln*	L
(7)	Oral Cavity	W	41	PD	P	L	A/C*	Asn/Asp*	Gln/Lys*	Gln/Arg*	L
(8)	Oral Cavity	W	60	CR	P	H	C/C	Asp/Asp	Lys/Lys	Gln/Arg	H
(9)	Oral Cavity	M	44	CR	P	H	C/C	Asn/Asn	Gln/Gln	Arg/Arg	H
(10)	Oral Cavity	M	60	ED	P	L	C/C	undet.	undet.	undet.	L
(11)	Oral Cavity	W	59	PD	P	L	A/C	Asn/Asp	Gln/Lys	Arg/Arg	H
(12)	Oral Cavity	W	55	CR	Undet.	Undet.	A/C	Asp/Asp	Lys/Lys	Gln/Arg	Undet.
(13)	Oropharynx	M	57	CR	P	H	C/C	undet.	undet.	undet.	H
(14)	Oropharynx	W	36	CR	P	L	C/C	Asn/Asp	Gln/Gln	Gln/Arg	H
(15)	Oropharynx	M	59	CR	P	L	A/C	Asn/Asp	Gln/Lys	Gln/Arg	L
(16)	Oropharynx	W	55	CR	N	H	A/C	Asn/Asp	Gln/Lys	Arg/Arg	H
(17)	Oropharynx	M	69	PR	P	Undet.	A/C	Asn/Asn	Gln/Lys	Gln/Arg	Undet.
(18)	Oropharynx	M	46	CR	N	L	C/C	Asn/Asp	Gln/Lys	Arg/Arg	L
(19)	Oropharynx	M	73	PD	P	L	C/C	Asp/Asp^⋀^	Gln/Lys	Gln/Gln	L
(20)	Oropharynx	M	67	SD	P	H	A/A	Asn/Asn	Gln/Gln	Gln/Arg	L
(21)	Hypopharynx	M	46	CR	P	H	A/C	Asn/Asp	Gln/Lys	Gln/Arg	H
(22)	Hypopharynx	W	64	PR	N	H	C/C	Asp/Asp	Lys/Lys	Arg/Arg	L
(23)	Hypopharynx	M	56	ED	P	L	A/C	Asn/Asp^⋀^	Lys/Lys	Gln/Arg	L
(24)	Hypopharynx	W	56	PR	P	L	C/C*	Asn/Asp*	Gln/Lys*	Gln/Arg*	H
(25)	Larynx	M	55	CR	N	H	A/C	Asn/Asp	Gln/Lys	Gln/Arg	H
(26)	Larynx	M	68	PR	N	L	A/A	Asp/Asp	Lys/Lys	Arg/Arg	L
(27)	Larynx	M	60	ED	—	Undet.	—	—	—	—	Undet.
(28)	Larynx	M	42	PR	P	N	C/C	Asn/Asn	Gln/Lys	Gln/Gln	H
(29)	Larynx	M	76	CR	P	H	A/C	Asn/Asp	Gln/Gln	Gln/Arg	L
(30)	Larynx	M	74	ED	P	N	C/C	Asn/Asp	Gln/Lys	Arg/Arg	L
(31)	Larynx	W	46	SD	P	N	A/C	Asn/Asp	Gln/Lys	Gln/Arg	L
(32)	Larynx	M	74	ED	N	H	A/A^⋀^	Asn/Asp	Lys/Lys	Gln/Arg	L
(33)	Larynx	M	67	PD	N	H	C/C	Asp/Asp	Lys/Lys	Arg/Arg	L
(34)	Larynx	M	54	CR	P	N	C/C	Asp/Asp	Lys/Lys	Arg/Arg	L
(35)	Larynx	M	54	PR	P	Undet.	A/C	Asn/Asp	Gln/Lys	Gln/Arg	Undet.
(36)	Paranasal Sinuses	M	65	SD	P	H	C/C	Asp/Asp	Gln/Lys	Gln/Arg	H
(37)	Major Salivary Glands	M	74	PR	P	H	C/C	Asp/Asp	Gln/Lys	Gln/Arg	H

n = sample order number; M = man; W = woman; H = high; L = low; P = positive; N = negative; # = genotypes from tumor tissue or from matched peripheral blood and tumor tissue samples, unless otherwise specified; ^⋀^ = mismatched tumor/peripheral blood genotypes (tumor data are shown); * = peripheral blood data only; Undet. = undetermined by real time PCR.

**Table 5:** Incidence of excision repair genotypes in head and neck cancer patients. Peripheral blood (PB) and tumor tissue (TT) data.

	ERCC1 C8092A/														
	CD3EAP Q504K			ERCC2-312 Asn/Asp				ERCC2-751 Lys/Gln				XRCC1-399 Gln/Arg			
	(CAG/AAG)			(AAC/GAC)				(AAG/CAG)				(CAG/CGG)			
PB (*n* = 26)	C/C	12	(46.2%)	(G/G)	Asp/Asp	8	(30.8%)	(C/C)	Gln/Gln	4	(15.4%)	(G/G)	Arg/Arg	8	(30.8%)
	A/C	13	(50%)	(A/G)	Asn/Asp	13	(50%)	(A/C)	Lys/Gln	15	(57.7%)	(A/G)	Gln/Arg	15	(57.7%)
	A/A	1	(3.8%)	(A/A)	Asn/Asn	5	(19.2%)	(A/A)	Lys/Lys	7	(26.9%)	(A/A)	Gln/Gln	3	(11.5%)
TT (*n* = 33)	C/C	17	(51.5%)	(G/G)	Asp/Asp	12	(38.7%)	(C/C)	Gln/Gln	5	(16.1%)	(G/G)	Arg/Arg	12	(38.7%)
	A/C	12	(36.4%)	(A/G)	Asn/Asp	15	(48.4%)	(A/C)	Lys/Gln	16	(51.6%)	(A/G)	Gln/Arg	16	(51.6%)
	A/A	4	(12.1%)	(A/A)	Asn/Asn	4	(12.9%)	(A/A)	Lys/Lys	10	(32.3%)	(A/A)	Gln/Gln	3	(9.7%)
	Undet.	0				2				2				2	

Undet. = undetermined with real time PCR.
